# Lysophosphatidic Acid Signaling and microRNAs: New Roles in Various Cancers

**DOI:** 10.3389/fonc.2022.917471

**Published:** 2022-06-23

**Authors:** Mahdi Rafiyan, Mohammad Hassan Jafari Najaf Abadi, Seyed Saeed Tamehri Zadeh, Michael R. Hamblin, Mahboubeh Mousavi, Hamed Mirzaei

**Affiliations:** ^1^ School of Medicine, Kashan University of Medical Sciences, Kashan, Iran; ^2^ Student Research Committee, Kashan University of Medical Sciences, Kashan, Iran; ^3^ Department of Medical Biotechnology, School of Medicine, Mashhad University of Medical Sciences, Mashhad, Iran; ^4^ School of Medicine, Tehran University of Medical Sciences, Tehran, Iran; ^5^ Laser Research Centre, Faculty of Health Science, University of Johannesburg, Doornfontein, South Africa; ^6^ Department of Anatomy, Faculty of Medicine, Semnan University of Medical Sciences, Semnan, Iran; ^7^ Center for Biochemistry and Nutrition in Metabolic Diseases, Institute for Basic Sciences, Kashan University of Medical Sciences, Kashan, Iran

**Keywords:** lysophosphatidic acid, cancer, molecular mechanism, microRNA, lysophosphatidic acid receptor

## Abstract

A wide range of microRNAs (miRNAs) are coded for in the human genome and contribute to the regulation of gene expression. MiRNAs are able to degrade mRNAs and/or prevent the RNA transcript from being translated through complementary binding of the miRNA seed region (nucleotide 2-8) to the 3’-untranslated regions of many mRNAs. Although miRNAs are involved in almost all processes of normal human cells, they are also involved in the abnormal functions of cancer cells. MiRNAs can play dual regulatory roles in cancer, acting either as tumor suppressors or as tumor promoters, depending on the target, tumor type, and stage. In the current review, we discuss the present status of miRNA modulation in the setting of lysophosphatidic acid (LPA) signaling. LPA is produced from lysophosphatidylcholine by the enzyme autotaxin and signals *via* a range of G protein-coupled receptors to affect cellular processes, which ultimately causes changes in cell morphology, survival, proliferation, differentiation, migration, and adhesion. Several studies have identified miRNAs that are over-expressed in response to stimulation by LPA, but their functional roles have not yet been fully clarified. Since RNA-based treatments hold tremendous promise in the area of personalized medicne, many efforts have been made to bring miRNAs into clinical trials, and this field is evolving at an increasing pace.

## Introduction

Gene regulation is critical for maintaining homeostasis, and both embryonic development and adult functioning depend on its appropriate function. Gene regulation ensures that appropriate genes are translated at the appropriate time. Gene regulation is a complex process, and various mechanisms are involved (1). MicroRNAs (miRNAs) are one of the most important mediators of gene regulation (2–5). MiRNAs were first discovered in *Caenorhabditis elegans*, a worm from the nematode phylum (6). Further investigations showed that miRNAs were present in almost every eukaryotic species, including human cells (7). MiRNAs are small, evolutionarily conserved RNA sequences that inhibit protein production by binding to their target mRNA. The evolutionary conservation of many miRNAs underlines their importance in biological functions (8). MiRNAs can regulate at least 30% of protein-coding genes (9, 10). “The majority of miRNAs are transcribed from DNA sequences into primary miRNAs and processed into precursor miRNAs, and finally mature miRNAs (11). In most cases, miRNAs interact with the 3′ untranslated region (3′ UTR) of target mRNAs to induce mRNA degradation and translational repression (12). However, the interaction of miRNAs with other regions, including the 5′ UTR, coding sequence, and gene promoters, has also been reported. Under certain conditions, miRNAs can also activate translation or regulate transcription” (13). Due to their effect on protein production, they are essential in cell signaling and cell proliferation, and in diseases like cancer, they can act as a tumor-suppressor and prevent cancer, or act as an oncomiR and promote cancer progression (14). In addition to being present inside cells, they are also found in extracellular fluids like cerebrospinal fluid (CSF) and blood and can be used as biological markers for some diseases or disease states (15). Also, recent studies have suggested that extracellular miRNA can serve as intercellular signaling molecules and as facilitator molecules for viral entry into cells (16). Secretion of extracellular miRNAs from cancer cells can alter the translational profile of normal cells and promote tumor progression (17).

Phospholipids are the principal components of the cell membrane. They play a crucial role in cell signaling, inflammation, endocytosis, exocytosis, etc. (18–21). Lysophosphatidic acid (LPA, 1-acyl-sn-glycerol-3-phosphate) is a phospholipid derivative with a glycerol backbone, a free phosphate group, and a single fatty acyl chain. Unlike other phospholipids, it is water-soluble (22) and acts as a potent inducer of platelet aggregation, smooth muscle contraction, and changes in blood pressure (23–25). Despite these discoveries, its primary function was not revealed until several years later. In the 1990s, scientists showed that LPA could act as a growth factor by interacting with specific G-protein receptors (26). LPA was then the subject of much more research, and additional functions for this molecule were discovered. One discovery concerned the role of LPA in cancers. The interaction of LPA with G-protein coupled receptors provides signaling inputs for cell growth and proliferation and therefore has a prominent role in cancer progression and development (22). Autotaxin (ATX) (also known as ectonucleotide pyrophosphatase/phosphodiesterase 2) produces LPA, which acts as a facilitator for tumor invasion, neovascularization, and metastasis (27). This review aims to discuss the interactions between LPA, its receptors, signaling pathways, and miRNAs and the possible role of this interaction in the development of diseases, especially cancer. This will help us better understand cancer pathogenesis and develop improved therapies involving miRNAs against various diseases.

## Lysophosphatidic Acid Structure and Biological Function

Lysophosphatidic acid (LPA, mono-acylglycerol-3-phosphate) is a phospholipid containing glycerol, a single fatty acid chain, and phosphate in its structure. Despite its relatively simple structure, it can cause significant changes within cells (28). It can be produced in two ways. Firstly, diacylglycerol can be phosphorylated by the action of diacylglycerol kinase to form phosphatidic acid (PA). Alternatively, phosphatidylcholine can be cleaved by the action of phospholipase D also to form PA. PA is converted to LPA in the next step by phospholipase A (29). This pathway is used chiefly for intracellular LPA production (30). Secondly, phospholipids are converted to lysophospholipids by phospholipase A2, and then lysophospholipase D cleaves it to LPA. This pathway is mainly used for extracellular LPA production (31).

**Figure 1:**
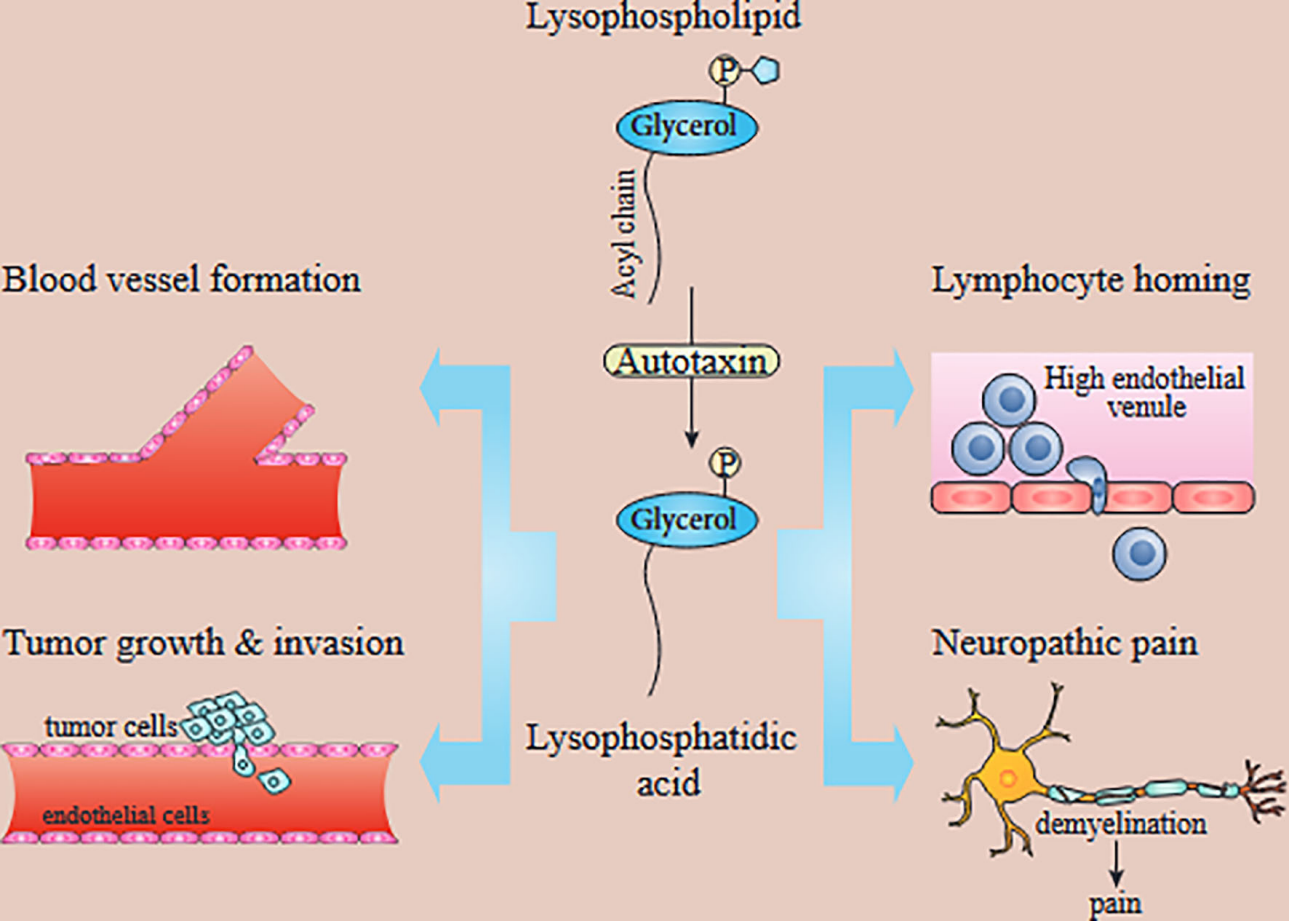
*In vivo* functions of the ATX/LPA axis. LPA produced by ATX has been implicated in blood vessel formation, neuropathic pain, lymphocyte homing, and tumor growth and metastasis. Autotaxin-produced lysophosphatidic acid is involved in tumor development, angiogenesis, metastasis, lymphocyte homing, and neuropathic pain [39–41]. While LPAR1 is known to promote tumor metastasis and neuropathic pain, the exact LPA receptors contributing to other processes are unknown.

Initially, the biological effects of LPA were not considered particularly important, with limited effects on smooth muscle contraction, platelet aggregation, and regulating blood pressure. Some of the LPA functions were rapid, like gap-junction closure, cell motility, or morphological changes, while others were more long-term, including wound healing, increased cell viability, etc. However, in the 1990s, scientists discovered that the LPA could behave as a growth factor molecule and trigger signaling *via* members of a specific type of G-coupled protein receptor family (22, 32). Also, LPA is a major serum component (but not plasma) and can increase cell survival and migration. Additionally, LPA is found in follicular fluid, saliva, and seminal fluid (22, 33–35). LPA concentrations are correlated with some types of cancers. For example, the Serum levels of LPA in pancreatic cancer patients were substantially higher than in healthy control (36). There are several studies indicating that plasma LPA is a promising diagnostic marker for ovarian cancer (37, 38). Autotaxin (ATX, ectonucleotide pyrophosphatase/phosphodiesterase 2, NPP2, or ENPP2) is an essential enzyme with several biological roles (**Figure 1**). Tanaka et al. showed the essential role of ATX in blood vessel formation (42). Another physiological role of ATX is in regulating immunity. ATX is expressed in the high endothelial cells (HEC) of high endothelial venules (HEV). Chemokine-activated lymphocytes bind to ATX, leading to LPA formation, which probably affects chemokinesis (43, 44). In addition to the essential physiological role of ATX in the body, several studies have confirmed its involvement in tumor progression, tumor invasion, and metastasis (32). Because ATX produces LPA from lysophosphatidylcholine, this was considered to explain the involvement of ATX in cancer (45, 46). LPA signaling takes place through G-coupled protein receptors as first reported in 1989 (26). Since then, six separate receptors that are specific for LPA signaling have been recognized (LPAR1-LPAR6). Each of these different receptors has its own tissue distribution and particular role and triggers different pathways. For example, LPAR2 has the highest expression in leukocytes. While the expression of LPAR5 is maximal in the spleen and in the same time it is not expressed in the liver (47). However, all the functions of some of these receptors have not been fully identified. LPAR1 is necessary for brain development, cancer metastasis, renal and lung fibrosis, etc. (48–50). LPAR2 is involved in smooth muscle cell migration, the progression of colorectal cancer, etc. (51–53). LPAR3 takes part in embryonic implantation (54–56). LPAR4 is probably involved in the pathogenesis of diseases like ovarian cysts. LPAR5 seems involved in thyroid malignancies (57–59), while LPAR6 is involved in hair growth (60, 61). In another study on pancreatic cancer, receptors of LPA (LPAR1, LPAR2, and LPAR3) are reported to be expressed significantly in PANC-1 cells. Following LPA treatment, focal adhesion kinase (FAK) and paxillin are tyrosine phosphorylated. Moreover, LPA can also increase the motility of PANC-1 cells through affecting the translocation of paxillin and FAK from cytoplasm to the adhesions of cell periphery (62). Leve et al. has reported while 10 μM of LPA cannot change the pattern of invasion, migration, and growth, it can affect cell cycle progression and proliferation in HCT-116 cells. Furthermore, LPA can increase the activation of STAT3 and Rho. Meanwhile, inhibition of STAT3 and ROCK promotes the LPA-induced expression of cyclins B1, E1, and A2 (63).

## MicroRNAs and Lysophosphatidic Acid in Various Cancers

**Table 1** lists various miRNAs involved with LPA and its signaling pathways in various cancers. Cancer is a significant cause of morbidity and mortality. According to the WHO, cancer has the most considerable effect on Disability-Adjusted Life Years (DALYs) among the top 20 causes of disease. Moreover, cancer imposes a tremendous cost on health care systems and is the second leading cause of death. Several mechanisms have been described to explain cancer initiation and progression, and many environmental and genetic factors are involved in its pathogenesis. One factor that has been recently studied is the role of miRNAs in cancer. miRNAs can alter the expression of oncogenes and tumor suppressor genes and thus can either promote or suppress cancer. Because, as mentioned above, LPA and its signaling pathway are important in cancer development, researchers have tried to discover the role of LPA in different cancers. Taken together, miRNAs, LPA, and their interactions could represent a potential target for cancer therapy.

**Table 1:** Various miRNAs have been reported to be involved with LPA and its signaling pathways in various cancers.

Disease	MicroRNA	Effect	Target	Model	Cell line	Ref
Gastric cancer	miR-501-5p	Increased proliferation and migration of GC cells by LPAR1 down-regulation	LPAR1	*In vitro*, Human	BGC823, MKN28, MGC803, SGC790	[64]
Ovarian cancer	miR-30c-2-3p	LPA increased the expression of both ATF3 mRNA and miR-30c-2-3p in ovarian and renal cancer cells. However, miR-30c-2-3p binds to ATF3 mRNA and inhibits its translation./LPA upregulated miR-30c-2-3p, which suppressed BCL9 expression and inhibited proliferation.	ATF3/BCL9	*In vitro*	SKOV-3, OVCAR-3/OVCAR-3, SKOV-3, HeyA8	[65, 66]
Osteosarcoma	miR-340-5p	MiR-340-5p targeted 3′ end of LPAATβ. Reduction of miR-340-5p increased the expression of LPAATβ.	LPAATβ	*In vitro*	MG-63, Saos-2	[67]
Kaposi Sarcoma	miR-K10a, miR-K4-3p (miRs encoded by Kaposi sarcoma-associated herpesvirus)	miRNAs of KHSV targeted ROCK2 and thus decreased the expression of ICAM1 mediated by LPA.	ROCK2	*In vitro*	Primary human umbilical vein endothelial cells (HUVECs)	[68]
Breast Cancer	miR-21	LPA upregulated miR-21 *via* LPAR1/PI3K/ZEB1 pathway.	PTEN, PDCD4, SPRY2	*In vitro*	MDA-MB-231, Hs578T	[69]
Malignant tumors	miR-489-3p	MiR-489-3p inhibited MEK1 and proliferation. In the presence of Autotaxin, this effect was reversed, and miR-489-3p increased the MEK1 level.	MEK1	*In vivo*		[70]
Cancer	miR-101-3p	miR-101-3p targeted ATX mRNA and inhibited ATX expression	ATX	*In vitro*	HT29, HCT116	[71]
Gastric cancer	miR-146a	NF-κB activation *via* LPA signaling is inhibited by miR-146a. miR-146a reduced cytokines and growth factors involved in tumor progression and monocyte attraction.	CARD10, COPS8	*In vivo*, Human		[72]
Osteosarcoma	miR-24	miR-24 targeted LPAATβ, and reduced proliferation.	LPAATβ	*In vitro* Human	MG63, 143B, hFOB1.19	[73]
Ovarian cancer	miR-15b	miR-15b represses the proliferation and drives the senescence and apoptosis of ovarian cancer cells through the suppression of LPAR3 and the PI3K/Akt pathway	LPAR3, Bcl-2	*In vitro* Human	ES-2, Caov-4, SKOV-3,OVCAR-3,OV-1063	

Sudeepti et al. showed that miR-489-3p could suppress MEK1 (mitogen-activated protein kinase), an enzyme that phosphorylates and activates mitogen-activated protein kinase encouraging tumor development and progression. Ironically, adding miR-489-3p to the cells that express ATX continuously increased the MEK1 activity. These results suggest that ATX can alter the function of miR-489-3p (70). Wang et al. investigated the role of miR-101-3p in cancer development. Their results suggested that miR-101-3p inhibited the expression of ATX, which is a critical factor in cancer progression. Additionally, they showed that adding LPA could abrogate this miRNA’s tumor suppressor activity (71).

### Breast Cancer

Excluding non-melanoma skin cancer, breast cancer (BrCA) is the most common cancer in women and has significant mortality (74, 75). Several pathophysiological risk factors, such as estrogen, progesterone, hormone receptors, etc., affect the patient’s prognosis and the tendency to metastasize (76–78). LPA and miRNAs are also involved in the pathophysiology of this cancer. LPA activates LPAR1, which activates 74 different genes that are important in tumor progression, invasion, and metastasis, for example, vimentin (VIM) (69, 79). In their study, David and his colleagues figured out that the LPA induced early gene expression in three kinds of unrelated cancer cell lines expressing various types of LPA receptors (79). Through the genes which were upregulated *via* LPA, only in the cells where LPAR1 was expressed, they proved by ELISA and q-PCR that Ki16425 and Debio0719 (LPAR1-3 antagonists) impeded the HB-EGF (heparin-binding EGF like growth factor) up-regulation. The HB-EGF mRNA upregulation and downregulation were evaluated by the human breast cancer cell lines MDA-B02/LPAR1 and MDA-B02/shLPAR1, respectively. Clinically, they measured the HB-EGF and LPAR1 levels through q-PCR in the primary tumors of 234 breast cancer patients who participated in a cohort study, and also found that their breast tumors significantly expressed high levels of HB-EGF. In their study, they evaluated the expression of HB-EGF mRNA in xenograft prostate tumors in mice injected with PC-3 cells and then went under five days of treatment with Ki1645. Their data revealed that HB-EGF could be utilized as a novel factor to demonstrate the LPAR1 activations in patients who were administered by anti-LPAR1 agents (79).

Sahay et al., by evaluating the 1488 human breast tumor data, revealed that the most correlated gene with LPAR-1 [encoding LPAR1] was the transcription factor ZEB1. Their data from three different basal cell lines proved that LPA induced the expression of ZEB1 and its modulation *via* the LPAR1/PI3K (Phosphatidylinositol-3-Kinase) pathway. Further analysis by RT-PCR and DNA microarray also showed that LPA, through the LPA1/PI3K/ZEB1-dependent mechanism, increased the expression of miR-21, one of the most well-known oncomiRs. Interestingly, utilizing the miR-21 inhibitor or silencing LPAR1 or ZEB-1 completely prevented the LPA-induced cell migration and also prevented invasion and tumor cell bone colonization entirely *in vitro* and *in vivo*, respectively. Finally, their results showed a new LPA-induced molecular pathway that targets LPAR1 in basal breast cancer patients (69).

### Ovarian Cancer

Ovarian cancer is another common malignancy and the eighth cause of cancer mortality among women (80). Ovarian cancer has several subtypes, each related to a specific type of cell resident within the ovary. These cell types include epithelial, serous, endometroid, clear cell, and mucinous. Recent investigations have shown that miRNAs, in common with many other cancers, are involved in ovarian cancer. MiR-15b is a member of the miR-15/16 family and is involved in cancers such as ovarian, gastric, glioma, and cerebral ischemic disease (81–83). MiR-15b regulates apoptosis by binding to the B cell lymphoma-2 (Bcl-2) 3’-UTR, which is highly expressed in cancer cells, hepatic cells, and mesenchymal stem cells (MSC) (84–86). In addition, miR-15b can interact with LPAR3 and the PI3K/Akt pathway. However, the effect of this miRNA is different from other miRNAs discussed above. MiR-15b can repress proliferation, migration, and invasion of ovarian cancer cells by binding to LPAR3 mRNA and inhibiting Bcl-2 and the PI3K/Akt pathway. Thus, this miRNA exerts an anti-tumorigenic effect (87).

MiR-30c-2-3p is another miRNA involved in ovarian cancer through LPA signaling and is increased in ovarian and renal cancer cells (66, 88). The lysophosphatidic acid signaling induces miR-30c-2-3p, which is correlated with the down-regulation of oncogenic mRNA like the BCL-9 transcript, and it could be so valuable in ovarian cancer (65).

ATF3 is a member of the molecular hub that belongs to the ATF/CREB (cyclic AMP response element-binding) family that plays an integral role in inducing the proliferation abilities of malignant cells. ATF3 expression is low, but the genotoxic agents, physiological stresses, and cytokines can increase its expression (89). The assessment of primary ovarian tumor gene expression revealed that the expression of ATF3 mRNA is directly correlated with symptoms of depression in patients suffering from ovarian carcinoma (90, 91). So these findings reveal the functional role of ATF3 in primary ovarian carcinoma (92). However, ATF3 has paradoxical effects on the different kinds of cancer. It has pro-tumorigenic effects, and on the other hand, it also has anti-tumorigenic effects. For instance, the role of ATF3 in bladder cancer is to suppress the metastasis abilities, but in cutaneous squamous cell carcinoma, it provokes the oncogenic effects (93). Moreover, in some types of ovarian cancer, ATF3 promotes the apoptosis mechanism, but in others, it is associated with poor prognosis outcomes (94).

According to the findings, miR-30c-2-3p inhibited the expression of transcription factors such as BCL9, Cyclin E1, X-box binding protein-1, and HIF2A, as well as the NF-kB signaling pathway and an adaptor protein (65, 95, 96). Part of the transcription repression mechanism for ATF3 is correlated with histone deacetylases and miR-494. However, it still needs more studies to explain the complete mechanism and elements involved with ATF3 regulation (97, 98).

The findings indicate that miR-30c-2-3p targets ATF3 mRNA. The bioinformatics techniques, luciferase assays, qRT-PCR, and immunoblotting revealed the supervisory pathway between the miR-30c-2-3p and ATF3. Lysophosphatidic acids can stimulate ATF3 and miR-30c-2-3p expression, as the highest expression rate occurs after one hour and disappears after 8 hours in ovarian cancer cells. Specific mutations introduced into the predicted interaction site between miR-30c-2-3p and the ATF3 3´-UTR diminish the luciferase signal. Additionally, after lysophosphatidic acid stimulation, the existence of anti-miR-30c-2-3p enhanced ATF3 mRNA and protein. These findings indicated that the expression of miR-30c-2-3p was increased in the presence of LPA, leading to increased proliferation in malignant cells (66). They also showed that miR-30c-2-3p could bind to ATF3 mRNA and inhibit its translation.

### Gastric Cancer

Gastric cancer is the fifth most common cancer in men and the third most common cause of cancer (99). Like other cancers, several pathophysiologic factors are essential in the progression of gastric cancer, such as inflammation, nuclear factor-κB (NF-κB) activation, immune suppression, etc. NF-κB is involved in cell proliferation and can be activated by G-protein coupled receptors (GPCR). Caspase recruitment domain-containing protein 10 (CARD10) and COP9 signalosome complex subunit 8 (COPS8) are two proteins involved in GPCR-mediated activation of NF-κB. LPA can activate the NF-κB pathway *via* these proteins and cause tumor progression. However, CARD10 and COPS8 are direct targets of miR-146a, and over-expression of this miRNA can inhibit the activation of NF-κB *via* LPA, reduce cytokine and growth factor secretion and inhibit monocyte attraction. MiR-146a probably has an essential role in NF-κB regulation as it is increased in most gastric cancers (72).

MiR-501-5p is another miRNA involved in the progression of gastric cancer (64).Ma et al. propose the study to evaluate the role of miR-501-5p in GC cell lines and explore its function and expression in human gastric cancer cell lines (64). By qRT-PCR, their data revealed that miR-501-5p expression increased in GC cell lines and carcinoma tissues. Their further analysis by cell migration assay and cell counting Kit-8 colony formation showed a direct correlation between the miR-501-5p downregulation and alleviated the GC cell migration and proliferation abilities. Moreover, they figured out that by silencing the miR-501-5p expression in GC cell lines, the cell cycle arrest in G2 and its apoptosis mechanism were provoked. The extra analysis by dual-luciferase reporter gene assay and Western blot analysis showed that the target of miR-501-5p was lysophosphatidic acid reporter-1 and reduced its expression in GC cell lines. Authors suggested that miR-501-5p down-regulated LPAR1 expression could promote cell proliferation and migration (64).

### Osteosarcoma

Osteosarcoma is the most common primary malignant bone tumor among children and adolescents (100). Despite its frequency, the precise mechanisms of osteosarcoma cell proliferation remain obscure. Different miRNAs are involved in osteosarcoma. Over-expression of miR-34a repressed tumor growth and metastasis of osteosarcoma, probably *via* c-Met down-regulation (101). A similar effect was caused by miR-125b, but this time through suppression of STAT3 (102). Mir-199a-3p is a regulator of cell proliferation and migration and is down-regulated in osteosarcoma (103). Mir-143 is involved in osteosarcoma by reducing cell viability, increasing apoptosis, and repressing tumorigenicity through targeting Bcl-2 (104). LPA is another molecular factor involved in osteosarcoma. Some studies have suggested that the increased expression of lysophosphatidic acid acyltransferase b (LPAATβ) promotes osteosarcoma proliferation. One of the targets of miR-24 is LPAATβ, which reduces the expression of LPA (73).

Song et al. discovered a link between osteosarcoma cell proliferation and increased LPAAT-expression levels. The increased expression of miR-24 in osteosarcoma cells resulted in the downregulation of LPAAT-expression. Overexpression of miR-24 reduced osteosarcoma cell proliferation while increasing LPAAT-activity in osteosarcoma cells (73). The other microRNA whose expression was reduced in osteosarcoma was miR-340-5p. MiR-340-5p functions similarly to miR-24 in that it targets LPAATβ. An additional study from Song et al. showed that miR-340-5p powered the cisplatin (CDDP)-induced cell death mechanisms. MiR-340-5p overexpression led to a decline of the IC50 of CDDP and induced the apoptosis of CDDP-resistant MG-63 and Saos-2 cells. Also, miR-340-5p reduced the MDR-1 and MRP-1 levels. They dug deeper into the mechanism of action of miR-340-5p in CDDP-induced cell death. Further evaluation and analysis through the online program Targetscan detected the miR-340-3p potential target in lysophosphatidic acid acyltransferase (LPAATβ). According to luciferase reporter experiments, miR-340-5p binds to the 3′UTR of LPAATβ and stimulates its degradation in both MG-63 and Saos-2 cells. Silencing LPAATβ decreased the IC50 of CDDP and increased apoptosis in CDDP-resistant MG-63 and Saos-2 cells, confirming miR-340’s influence on CDDP-induced cell death. Finally, their findings revealed that miR-340-5p increased CDDP sensitivity *via* LPAATβ targeting (67).

### Kaposi Sarcoma Virus

Kaposi’s sarcoma-associated herpesvirus (KSHV, human herpesvirus 8) is a herpes virus family member and induces a low-grade vascular tumor called Kaposi’s sarcoma. Usually, these lesions are found in mucocutaneous sites like the mouth. However, the disease can also occur in internal organs. This virus can express miRNAs (which is rare among viruses), and these miRNAs may be necessary for the suppression of the immune system (105). Previous research has shown that ROCK2 is involved in a pro-inflammatory pathway induced by lysophosphatidic acid (LPA), which results in the up-regulation of intercellular adhesion molecule 1 (ICAM1) on the surface of endothelial cells (106). ICAM1 binds to lymphocyte function-associated antigen 1 (LFA-1) and promotes leukocyte recruitment and transmigration. Interestingly, ICAM1 is downregulated from the cell surface and destroyed by the KSHV lytic protein, K5, in a well-described process, which might reduce helper T cell recruitment (107–109). As well as a reduction in ICAM1 expression during latent *de novo* endothelial cell infection (110). Gallaher et al. showed that the LPA- induced intercellular adhesion molecule 1 (ICAM1), which is vital for the proper function of the immune system against pathogens, was decreased by KSHV miRNAs, including miR-K4-3p and miR-K10a (68).

## Conclusion

The role of miRNAs in different diseases and normal bodily functions is now widely appreciated. MiRNAs affect gene expression, and thus they can change the fundamental phenotype of the cell. LPA signaling is important in many diseases and for the body’s normal functioning. Both of these molecules (LPA and miRNAs) can interact, and the inhibition of one can affect the others. LPA, LPA receptors, and their related signaling pathways have become a primary target for drug discovery efforts (111, 112).

Similarly, microRNA-based therapeutics are under broad investigation by pharmaceutical companies, and several candidates have entered clinical trials (113). Therefore the emerging interactions between these two important classes of biomolecules should be considered an important part of these pharmaceutical studies. Understanding the role of each of these molecules in turn, with an emphasis on how they can each affect the other, is becoming more vital to future efforts in drug development, especially for cancer but also for other diseases.

## Author Contributions

HM and MM were involved in the conception, design, statistical analysis, and drafting of the manuscript.MR, MHJ, SSTZ, and MRH contributed to data collection and manuscript drafting. All authors approved the final version for submission.

## Conflict of Interest

The authors declare that the research was conducted in the absence of any commercial or financial relationships that could be construed as a potential conflict of interest.

## Publisher’s Note

All claims expressed in this article are solely those of the authors and do not necessarily represent those of their affiliated organizations, or those of the publisher, the editors and the reviewers. Any product that may be evaluated in this article, or claim that may be made by its manufacturer, is not guaranteed or endorsed by the publisher.
